# The Baltimore Family Health Journal

**Published:** 1890-11

**Authors:** 


THE BALTIMORE FAMILY HEALTH JOURNAL. Published every month. Price, $1.00 per year. Edited and published by Flora A. Brewster, M. D., and Cora B. Brewster, M. D., 1027 Madison Avenue, Baltimore, Maryland.

This periodical of about twenty-four pages a month is edited and published exclusively by ladies; the editorial staff, including, besides the above publishers, Professor Elizabeth J. French. It is intended for the home circle, and to instruct the family in those simple rules of conduct and of hygiene which are of so much importance in taking care of the health. Thus we find an excellent article upon self-control, which of itself will prevent much sickness; an article upon cooking, impure air in dwellings, etc. We cordially recommend this journal. W. M. J.

